# Tuberculosis infection control practices in a high-burden metro in South Africa: A perpetual bane for efficient primary health care service delivery

**DOI:** 10.4102/phcfm.v10i1.1628

**Published:** 2018-05-30

**Authors:** Michelle C. Engelbrecht, Gladys Kigozi, Andre P. Janse van Rensburg, Dingie H.C.J van Rensburg

**Affiliations:** 1 Centre for Health Systems Research and Development, University of the Free State, South Africa; 2 Health and Demographic Research Unit, Department of Sociology, Ghent University, Belgium; 3 Department of Political Science, Stellenbosch University, South Africa

## Abstract

**Background:**

Tuberculosis (TB) prevention, including infection control, is a key element in the strategy to end the global TB epidemic. While effective infection control requires all health system components to function well, this is an area that has not received sufficient attention in South Africa despite the availability of policy and guidelines.

**Aim:**

To describe the state of implementation of TB infection control measures in a high-burden metro in South Africa.

**Setting:**

The research was undertaken in a high TB- and HIV-burdened metropolitan area of South Africa. More specifically, the study sites were primary health care facilities (PHC), that among other services also diagnosed TB.

**Methods:**

A cross-sectional survey, focusing on the World Health Organization levels of infection control, which included structured interviews with nurses providing TB diagnosis and treatment services as well as observations, at all 41 PHC facilities in a high TB-burdened and HIV-burdened metro of South Africa.

**Results:**

Tuberculosis infection control was poorly implemented, with few facilities scoring 80% and above on compliance with infection control measures. Facility controls: 26 facilities (63.4%) had an infection control committee and 12 (29.3%) had a written infection control plan. Administrative controls: 26 facilities (63.4%) reported separating coughing and non-coughing patients, while observations revealed that only 11 facilities (26.8%) had separate waiting areas for (presumptive) TB patients. Environmental controls: most facilities used open windows for ventilation (*n* = 30; 73.2%); however, on the day of the visit, only 12 facilities (30.3%) had open windows in consulting rooms. Personal protective equipment: 9 facilities (22%) did not have any disposable respirators in stock and only 9 respondents (22%) had undergone fit testing. The most frequently reported barrier to implementing good TB infection control practices was lack of equipment (*n* = 22; 40%) such as masks and disposable respirators, as well as the structure or layout of the PHC facilities. The main recommendation to improve TB infection control was education for patients and health care workers (*n* = 18; 33.3%).

**Conclusion:**

All levels of the health care system should be engaged to address TB prevention and infection control in PHC facilities. Improved infection control will address the nosocomial spread of TB in health facilities and keep health care workers and patients safe from infection.

## Background

In South Africa, primary health care (PHC) facilities are the entry point for diagnosis, treatment and management of tuberculosis (TB) and multidrug-resistant TB (MDR TB). With a TB incidence of 834 cases per 100 000 population, a 61% HIV co-infection rate^1^ and 18.8% HIV prevalence among adults aged 15–49 years,^2^ the health care system is overwhelmed in dealing with the co-epidemic. As TB transmission frequently occurs before an accurate diagnosis is made, appropriate infection control is essential.^3,4^ Virtually every country in the world – regardless of their TB incidence – has reported the spread of TB in health care settings to both patients and health care workers (HCWs).^5^ The responsibility rests with HCWs, particularly managers, to ensure the implementation of appropriate TB prevention and infection control measures in health care facilities.^3,4^ In support of this, major international and national agencies have issued guidelines for minimising TB exposure and subsequent infection in health care facilities.^6,7^

World Health Organization (WHO)^7,8,9^ recommends four levels of TB infection control – an overarching managerial and facility-level encompassing administrative and environmental controls, and personal respiratory protection. While WHO guidelines have permeated local policy content,^6,10^ South Africa has a record of developing progressive policies and programmes only to see them failing to trickle down to the service delivery level.^11,12,13^ Over the past 6 years, several studies have highlighted the poor implementation of TB infection control measures and practices at health facilities in South Africa.^14,15,16,17,18,19,20,21,22,23,24,25^ However, this growing body of research has mainly focused on hospital settings, with a limited number of studies undertaken at PHC facilities. Although limited, available research does suggest a pressing need for evidence and interventions to prevent TB transmission in PHC settings^19,21,25^ – including foci on policy content and implementation, TB prevention and infection control strategies, and knowledge and behaviour of HCWs and patients regarding prevention. This article aims to describe the state of implementation of TB infection control measures in a high-burden metro in South Africa. More specifically, the TB incidence, 686 per 100 000 population, and HIV prevalence among 15–49-year-old women attending antenatal care, 30.4%, was higher in the metro than the country as a whole (593 per 100 000 population and 29.7% respectively).^26,27^

## Methods

### Design, population and setting

A cross-sectional survey, with researcher-administered questionnaires for nurses providing TB services as well as physical observations, was undertaken at all 41 PHC facilities in a high TB-burdened and HIV-burdened metro, one of seven metropolitan areas, in South Africa. Tuberculosis nurses were purposively selected to participate in the survey, as they were seen to be the most knowledgeable about the state of TB infection control practices in PHC facilities. Of the 41 TB nurses who participated in the study, the majority (90.2%) were female; the average age was 49.93 years (standard deviation [s.d.] 8.577); slightly more than half had a secondary education (*n* = 22; 53.7%), 10 had a tertiary diploma (24.4%) and 9 had a tertiary degree (22.0%). The TB nurses had worked an average of 5.95 years (s.d. 6.9) at their current facility.

### Data collection and analysis

A review of the literature and existing TB prevention and infection control questionnaires^28,29,30^ informed the development of a facility assessment tool to interview nurses. The facility assessment tool addresses the WHO levels of infection control (i.e. facility, administrative, environmental and personal protective equipment) for TB in health care settings. The questionnaire was pilot-tested at PHC facilities falling outside of the study area and revised accordingly. In addition, a structured observation schedule was developed to verify the implementation of TB infection control practices. Data collection took place between October and November 2015.

The data were captured, cleaned and analysed in IBM SPSS Statistics 23. Data were described using frequency counts and percentages for categorical variables and means and standard deviations for continuous variables. Composite scores were calculated for the four levels of infection control (both self-reported by the TB nurse and observed by the researchers) – facility, administrative, environmental and personal protective equipment – to determine how compliant facilities were with these controls. While facilities should comply with 100% of all infection control measures, it was decided to use 80% compliance as the cut-off point for this study.

### Ethical considerations

Ethical clearance was obtained from the Ethics Committee of the Faculty of Health Sciences, University of the Free State (ECUFS No92/2013), and the study was authorised by the Provincial Department of Health. All participants completed consent forms prior to participating in the research.

## Results

### Background and demographic information

All 41 surveyed PHC facilities diagnosed TB in patients, and of these 39 provided monthly follow-up treatment and care for TB. The facilities were mainly staffed by professional nurses, with an average of 5.6 professional nurses per facility (s.d. 8.8). Three of the 41 TB nurses indicated that they had not received any training on TB diagnosis or management and just less than half (*n* = 18; 44.0%) had attended TB infection control training.

### Managerial and facility levels of tuberculosis infection control

Of the 26 facilities with an infection control committee (63.4%), 10 had met within 4 weeks prior to the interview (38.5%) and only five facilities were able to produce the agenda and minutes of their latest infection control meeting. Twelve facilities (29.3%) reported having a TB infection control plan, although only five could produce the plan. Provincial (*n* = 11; 26.8%), district (*n* = 12; 29.3%) and sub-district (*n* = 14; 35.0%) support was largely lacking for TB infection control (see Table 1).

**TABLE 1:** Reported facility-level infection control (*N* = 41).

Reported	*n*	%
Facility has an infection control committee†	26	63.4
Infection control committee met within past 4 weeks†	10	38.5
Provincial representative supporting infection control	11	26.8
District representative supporting infection control	12	29.3
Sub-district representative supporting infection control	14	35.0
HCWs are routinely trained on infection control†	13	31.7
TB infection control assessment conducted for the facility†	23	56.1

HCW, health care workers; TB, tuberculosis.

† , items summed to create a composite score for compliance with facility-level controls.

Overall, there was low compliance with facility control measures – the facility assessment found that five facilities (12.2%) scored 80% and above on compliance when a composite score was computed for the facility-level controls, while observations revealed that only three facilities (7.3%) scored 80% and above.

### Administrative-level controls

While self-reported good TB infection control practices were high, observations revealed a different picture (see Table 2). For example, 26 nurses (63.4%) reported that coughing and non-coughing patients were separated at their facilities, while observations revealed that only 11 facilities (26.8%) had separate waiting areas for (presumptive) TB patients. Furthermore, 30 nurses (73.2%) indicated that coughing patients were provided with masks; however, only three facilities had masks available for patients, and on the day of the observations only two of these facilities had coughing patients wearing masks.

**TABLE 2:** Reported and observed administrative-level infection control (*N* = 41).

Reported	*n*	%	Observed	*n*	%
Dedicated TB consulting rooms†	21	51.2	Not applicable	-	-
TB screening tool used†	41	100	TB screening tool	41	100
Patients coughing for more than 2 weeks tested for TB†	41	100	Not applicable	-	-
Contact tracing for TB patients†	33	80.5	Not applicable	-	-
TB health education in waiting areas†	40	97.6	Not applicable	-	-
Patients educated on TB infection control†	38	92.7	Not applicable	-	-
Patients educated on cough etiquette†	40	97.6	Not applicable	-	-
Coughing patients provided with tissues†	13	31.7	Tissues for coughing patients	7	17.1
Coughing patients provided with masks†	30	73.2	Masks for coughing patients Patients wearing masks	3 2	7.3 4.9
Coughing patients separated from non-coughing patients†	26	63.4	Not applicable	-	-
TB patients separated from the rest of the patients†	25	61.0	Separate waiting area for (presumptive) TB patients	11	26.8
Coughing patients prioritised (shorter waiting times)†	36	87.8	Not applicable	-	-
TB patients prioritised (shorter waiting times)†	32	78.0	Not applicable	-	-
Designated outside area for sputum collection†	41	100	Not applicable	-	-
Fast queue for sputum results†	37	90.2	Not applicable	-	-
Not applicable	-	-	Red plastic container for disposal of masks and tissues in waiting area†	3	7.3
Not applicable	-	-	Red plastic container for disposal of masks and tissues in consulting room†	37	90.2
Not applicable	-	-	‘Open window’ stickers†	35	85.4
Not applicable	-	-	‘Open door’ stickers†	10	24.4

Note: With regard to the administrative controls, 19 facilities (46.3%) scored 80% and above on compliance in the facility assessment, while actual observations showed that none of the facilities complied with 80% or more of the administrative controls that were investigated.

TB, tuberculosis.

† , items summed to create a composite score for compliance with facility-level controls.

### Environmental controls

Most facilities used open windows for ventilation (*n* = 30; 73.2%); however, on the day of the visit (in Spring) only 12 facilities (30.3%) had open windows in the consulting rooms and 9 facilities (22.0%) had open windows in the waiting areas (see Table 3).

**TABLE 3:** Reported and observed environmental-level infection control (*N* = 41).

Variables	*n*	%
**Reported**		
Designated person to open windows	28	68.3
UVG lights used in high-risk areas	0	0.0
Only open windows for ventilation	30	73.2
Open windows and fans available for ventilation	7	17.1
Extractor fans (whirly birds) available for ventilation	2	4.9
Air conditioning available for ventilation	1	2.4
TB nurse trained on air flow for infection control	22	53.7
**Observed**		
Open window register	12	30.0
Open windows in all consulting rooms	12	30.0
Open windows in all waiting areas	9	22.0

TB, tuberculosis; UVG, ultraviolet germicidal lights.

Overall, there was poor compliance with environmental controls at the facilities – facility assessments found that none of the facilities scored 80% and above on the composite score calculated for compliance with environmental controls, while observations showed that two facilities scored 80% and more on compliance with environmental controls.

### Personal protective equipment

Of the 39 respondents (95.1%) who had used disposable respirators, 5 reported that they did not know how often or when they should replace the respirators. The remaining respondents (*n* = 34; 87.2%) indicated that disposable respirators were replaced when it was dirty, wet or damaged (*n* = 14); once a month (*n* = 6); every 2–5 days (*n* = 4); daily (*n* = 4); after being in contact with an MDR TB patient (*n* = 2); twice a month (*n* = 2); after every use (*n* = 1); and one respondent never replaced it. Only 12 respondents (29.3%) had received training on respiratory protection and even fewer respondents had undergone a fit test for disposable respirators (*n* = 9; 22%) (see Table 4).

**TABLE 4:** Reported and observed use of personal protective equipment (*N* = 41).

Variables	*n*	%
**Assessed**		
Disposable respirators used by HCWs	39	95.1
Disposable respirators used by patients	3	7.3
Masks used by HCWs	9	22.0
Masks used by patients	34	82.9
Respiratory protection training conducted for HCWs	12	29.3
HCWs undergone fit test for respirator use	9	22.0
No protection used when observing a patient produce sputum	17	41.5
**Observed**		
Disposable respirators available	32	78.0
Nurses wearing disposable respirators	5	12.2

HCWs, health care workers.

Three facilities scored 80% and above on the composite score for compliance with personal protective equipment, while observations revealed that none of the facilities scored 80% and above for this level of infection control.

### Barriers to effective implementation of tuberculosis infection control practices in primary health care facilities

The most frequently reported barrier to implementing good TB infection control practices was lack of equipment (*n* = 22; 40%), such as masks, disposable respirators and appropriate waste disposal containers for tissues and masks. The structure and layout of the PHC facilities was also an obstacle to good infection control practices (*n* = 15; 27.3%), for example the lack of space for separate waiting areas for coughing and non-coughing patients and the location of the TB consulting room within the facility (e.g. the TB room was located at the back of the facility, which meant that coughing patients walked through the entire facility to reach the TB room). Further challenges included poor ventilation within the facilities (*n* = 11; 20%), poor cough etiquette of patients (*n* = 4; 7.3%) and poor staff attitudes towards TB infection control (*n* = 3; 5.5%).

### Recommendations to improve tuberculosis infection control practices at primary health care facilities

The main recommendation to improve TB infection control was education for patients and HCWs (*n* = 18; 33.3%). Further recommendations included addressing the poor physical structure and layout at PHC facilities and ensuring that there were designated TB consultation rooms and sputum collection areas (*n* = 14; 25.9%); ensuring that sufficient infection control resources, such as masks, disposable respirators, fans and UVG lights were readily available at PHC facilities (*n* = 9; 16.7%); sufficient nurses and community health care workers for TB management (*n* = 6; 11.1%); concerted efforts to keep windows and doors open (*n* = 4; 7%); and prompt screening of patients and HCWs (*n* = 3; 5.6%).

## Discussion and recommendations

Prevention is a key element in the strategy to end the global TB epidemic^1^ and an important component of prevention is infection control. However, this is largely neglected in South Africa despite the availability of policy and guidelines.^31^ Similar to other studies,^14,15,16,17,18,19,20,21,22,23,24,25^ we found poor adherence to TB infection control measures. Based on self-reports by the nurses, 16.5% of the facilities scored 80% and above on compliance with implementation of TB infection control measures. Actual observations were even more reason for concern, with only 3% of the facilities scoring 80% and above for implementation of TB infection control measures. This implies that HCWs and patients are largely unprotected from the spread of TB in PHC facilities, despite evidence^5^ that well-implemented infection control measures can reduce the spread of TB in health care facilities. This poses a serious risk for the PHC system in general and TB management and control in particular.

More specifically, at facility level, we found a lack of clinic infection control committees^19,21^ and plans,^3,19^ as well as inadequate support from sub-district, district and provincial managers for TB infection control. This is not surprising given the low rate of PHC supervisory visits in the metro.^32^ Administrative and environmental controls were also poorly implemented. For example, coughing patients were not separated from non-coughing patients^3,19,21^ and coughing patients were not provided with masks.^21,25^ Furthermore, windows were generally kept closed in TB consultation rooms and waiting areas.^19,21,25^ Poorly implemented administrative and environmental controls force HCWs to be responsible for their own safety by using personal protective equipment. However, the use of disposable respirators was also sporadic,^19,21^ and in most instances fit testing for disposable respirators was not done, although it ensures better protection if the facial seal is correctly fitted.^33^ As noted by Zelnick et al.,^22^ the use of personal protection that rests on individual discretion and behaviour is unlikely to be effective when there is stigma associated with the disease and unemployment rates are high – as in the case of South Africa.

The main barriers preventing the implementation of appropriate TB infection control measures were the layout of the clinics which did not allow for the separation of coughing patients, poor ventilation and the lack of basic resources such as masks and disposable respirators. It was recommended that TB infection control could be improved if facilities were renovated to allow for designated TB consultation rooms and separate waiting areas for coughing patients. However, physical changes to facilities are costly, and much of public health care infrastructure is run down and dysfunctional because of funding shortages, mismanagement and neglect.^34^ In order for PHC HCWs to effectively implement TB infection control, all health system components are required to function well: governance and stewardship, financing, infrastructure, procurement and supply chain management, human resources, health information systems, service delivery and finally supervision.^35^

Keeping with the hierarchy of TB infection control, simple, cost-effective measures should be prioritised when implementing TB infection control practices in PHC facilities. In this regard, administrative control measures are the first line of defence and should be prioritised, as the aim is to prevent staff and patients from being exposed to TB through rapid diagnostic investigation and treatment of presumptive TB. Environmental control measures form the second line of defence and complement administrative measures. At the lowest level of the hierarchy, it is recommended that personal protective equipment should be used together with administrative and environmental controls in situations where there is an increased risk of TB transmission.^7,8^

Therefore, the administrative level, as the first line of defence, should be prioritised. All PHC staff should be trained on cough etiquette and tasked with distributing masks and tissues to coughing patients. Facility managers and TB nurses require guidance on how best to use the space available at clinics to separate coughing and non-coughing patients. With reference to environmental controls, the second line of defence, TB nurses need training on airflow, and how to position themselves in the TB consultation room. All PHC staff and patients must be constantly reminded of the importance of natural ventilation to prevent TB transmission. This could be done through health education, ‘open window’ and ‘open door’ stickers, posters, etc. With regard to the use of personal protective equipment, PHC staff require information and training on disposable respirators, including fit testing of respirators and when to use and discard them. Finally, at the overarching managerial level, active involvement of provincial and district managers in supporting PHC facility managers and TB nurses is recommended. Integral to such support is regular supervisory visits and TB infection control assessments at facilities, individualised feedback and support to facilities regarding the state of their infection control implementation, and where necessary training and mentoring for all PHC facility staff on TB infection control.

Our study adds value to the growing body of evidence on TB infection control in low- and middle-income countries, given that South Africa, along with the other 22 high-burdened HIV and TB countries, does not systematically collect data on TB infection control implementation at a national level.^36^ Furthermore, while there are many studies investigating the implementation of TB infection control measures in health care facilities, the majority of these studies focus on hospital settings. Our study focused on the PHC setting, as it is the main entry point for screening, diagnosis and care of TB patients in South Africa. In addition, we did not only rely on self-reported implementation of TB infection control methods as respondents tend to provide socially desirable responses^37^ but also undertook observations at the facilities to verify reports by TB nurses.

However, as with all research, ours has limitations. The data were cross-sectional in nature, which means that we cannot infer strong causal claims. By only interviewing TB nurses, and not all PHC nurses, about infection control in their facilities we may have missed information. We did attempt to counter this by observing certain infection control practices at the facilities. Furthermore, it was beyond the scope of our study to explore in-depth reasons as to why HCWs did not implement appropriate infection control measures: qualitative enquiry and explanatory models are two possibilities for further work in this regard.

### Conclusion

TB infection control in PHC facilities remains problematic. A holistic approach is needed to address this challenge, and should target individuals, the environment and policy. At the individual level, HCWs need knowledge about TB infection control as well as support from managers and supervisors to implement effective practices. TB patients also need the knowledge to implement good infection control practices at home, health facilities as well as in the broader community. At the environmental level, infrastructural and layout changes are costly to implement, and as such HCWs should be encouraged and supported to implement simple, cost-effective measures, such as locating the TB consultation rooms close to an entrance, keeping windows open, and proper airflow. Furthermore, adequate supplies such as masks and disposable respirators should always be available at PHC facilities. Finally, at the policy level, the policies and guidelines for good infection control practices that protect both HCWs as well as patients from exposure to TB in health facilities should be workshopped with all PHC staff so that they understand the importance of and how to implement these guidelines.
